# Distribution and Risk of Cutaneous Leishmaniasis in Khyber Pakhtunkhwa, Pakistan

**DOI:** 10.3390/tropicalmed8020128

**Published:** 2023-02-20

**Authors:** Wasia Ullah, Tsai-Ying Yen, Sadaf Niaz, Nasreen Nasreen, Yu-Feng Tsai, Roger Ivan Rodriguez-Vivas, Adil Khan, Kun-Hsien Tsai

**Affiliations:** 1 Department of Zoology, Abdul Wali Khan University, Mardan 23300, Khyber Pakhtunkhwa, Pakistan; 2 Institute of Environmental and Occupational Health Sciences, College of Public Health, National Taiwan University, Taipei 100025, Taiwan; 3 Facultad de Medicina Veterinaria y Zootecnia, Campus de Ciencias Biologicas y Agropecuarias, Universidad Autonoma de Yucatán, Km 15.5 Carretera Mérida–Xmatkuil, Merida 97100, Yucatan, Mexico; 4 Department of Botany/Zoology, Bacha Khan University, Charsadda 24420, Khyber Pakhtunkhwa, Pakistan; 5 Department of Public Health, College of Public Health, National Taiwan University, Taipei 100025, Taiwan

**Keywords:** cutaneous leishmaniasis, *Leishmania*, clinico-epidemiology, geographic information systems (GIS), Khyber Pakhtunkhwa

## Abstract

Cutaneous leishmaniasis (CL) is a zoonotic infection caused by obligate intracellular protozoa of the genus *Leishmania*. This study aimed to investigate CL in Khyber Pakhtunkhwa, Pakistan and to estimate the risk of epidemics. Clinico-epidemiological data of 3188 CL patients were collected from health facilities in 2021. Risk factors were analyzed using the chi-square test. ArcGIS V.10.7.1 was applied for spatial analysis. The association between CL occurrence and climatic variables was examined by Bayesian geostatistical analysis. The clinical data revealed males or individuals younger than 20 years old were more affected. Most patients presented with a single lesion, and the face was the most attacked body part. CL was prevalent in the southern region in winter. A proportional symbol map, a choropleth map, and a digital elevation model map were built to show the distribution of CL. Focal transmission was predicted by inverse distance weighting interpolation. Cluster and outlier analysis identified clusters in Bannu, Dir Lower, and Mardan, and hotspot analysis suggested Bannu as a high-risk foci. Bayesian geostatistical analysis indicated that increasing precipitation and temperature as well as low altitudes were associated with CL infection. The study has provided important information for public health sectors to develop intervention strategies for future CL epidemics.

## 1. Introduction

Leishmaniasis is a zoonotic infection caused by obligate intracellular protozoa of the genus *Leishmania*. Over 20 species of *Leishmania* parasites have been linked to diseases in humans. Natural transmission of leishmaniasis is carried out by sandflies of the genus *Phlebotomus* in the Old World and *Lutzomyia* in the New World [1,2]. Hyraxes, canids, and rodents are common reservoirs, but some species of *Leishmania* are found to be anthroponotic [3]. After being injected into the host’s skin, *Leishmania* escapes host immune responses by infecting and proliferating in phagocytes, especially macrophages, and results in varied clinical manifestations with diverse prognoses [4,5]. Four clinical forms have been categorized, namely visceral leishmaniasis (VL), cutaneous leishmaniasis (CL), mucocutaneous, and post kala-azar dermal leishmaniasis (PKDL). Leishmaniasis is endemic in many tropical and subtropical regions, affecting more than 12 million people in at least 88 countries and leading to a morbidity and mortality loss of 2.4 million disability-adjusted life-years (DALYs) and nearly 70,000 deaths [6,7,8]. According to the World Health Organization (WHO), the estimated annual incidence of CL and VL have ranged from 600,000 to 1 million and 50,000 to 90,000, respectively. About 90% of both cutaneous and visceral leishmaniases have been reported from developing countries such as Afghanistan, Bangladesh, Brazil, Saudi Arabia, Syria, and Peru [9].

CL is the most common form of leishmaniasis and is characterized by one or several ulcers or nodules developing at the sites of the infectious sandfly bites. CL has been known in human history for centuries. It was mentioned in the Old World in the first century and in Ecuador and Peru in the New World from 400–900 AD [10,11,12]. Illness resembling CL was called ‘Aleppo evil’ in the Middle East and ‘Dehli boil’ in the Indian subcontinent in the 16th and 17th centuries, when protozoa were identified in skin lesions [13,14]. Different names, including Rose of Jericho, forest yaws, Baghdad sore, Uta, and Chiclero’s ulcer have been used to describe CL lesions [14]. CL continued to spread via the aid of travel and habitat expansion. Risk factors such as urbanization, migration, population surge and displacement, drug resistance, human modification of the environment, and new agricultural practices have been associated with the emergence of new foci. Infection caused by new species has also been documented [15,16,17,18].

In Pakistan, approximately 21,700–35,700 cases of CL are reported annually, and epidemics have occurred in Punjab (Multan), Baluchistan, and Khyber Pakhtunkhwa [19]. CL has been widespread in communities with low-income and in neglected areas due to their limited access to health care [20]. Moreover, the situation of leishmaniasis in Pakistan has been exacerbated. Nowadays, both VL and CL have been reported in numerous regions of the country, including Khyber Pakhtunkhwa [21]. Located in the north-west, Khyber Pakhtunkhwa has been one of the provinces most affected by CL [3]. The inflow of Afghan refugees resulted in the spread of CL to formerly non-endemic areas [22]. CL has also become an issue faced by the peacekeeping forces and soldiers posted in Afghanistan and the Federally Administered Tribal Areas (FATA) of Pakistan [23,24].

Geographic information systems (GIS) is a computer-based tool which has been widely applied to health science research [25]. As global positioning system (GPS) mobile devices have become popular, GIS and remote sensing have been increasingly adopted to determine the spatial epidemiology of vector-borne diseases, including malaria, dengue fever, leishmaniasis, etc. [26,27,28,29]. The distribution and spread of vector-borne diseases largely depend on the environmental requirement of vectors. Temperature, rainfall, urbanization, vegetation, and human society are known factors influencing vector behaviors [30]. Commercial GIS tools such ArcGIS incorporating statistical models in the software can not only be used for disease mapping for risk assessment but also can be used to estimate vector abundance and incidence by integrating data of climatic, demographic, and environmental factors [31,32]. Hence, control programs can be designed and implemented targeting a specific region based on local vector dynamics and their interactions with the hosts [33,34]. However, sandfly activities have not been constantly monitored in Pakistan despite the fact that a few studies have reported the environmental risk factors affecting sandfly populations [35,36,37]. Bayesian geostatistical analysis would provide a flexible approach to the availability of data, demonstrated by the identification of critical variables for disease transmission of malaria, schistosomiasis, and leishmaniasis [38,39,40,41]. The probabilistic likelihood-based framework of the Bayesian approach allows assimilation of available information, and the use of Markov chain Monte Carlo methods for data augmentation offers the chance to treat missing data [42].

The epidemiology of CL is changing in Pakistan, especially in the border lands in Khyber Pakhtunkhwa, and CL has emerged as a public health concern in past decades. However, lack of disease surveillance and vector data have limited the effectiveness of control efforts. This study aimed to illustrate a risk map of CL in Khyber Pakhtunkhwa. The epidemiology of CL was investigated based on clinical data. The spatial distribution of cases was analyzed by ArcGIS software, and the transmission efficacy, active zones, and coming epidemics throughout the study area were explored. Bayesian geostatistical analysis was applied to determine the environmental risk factors. The results provide an overview of CL in Khyber Pakhtunkhwa and would be helpful in developing control strategies for the disease in the region.

## 2. Materials and Methods

### 2.1. Study Area

**Figure 1:**
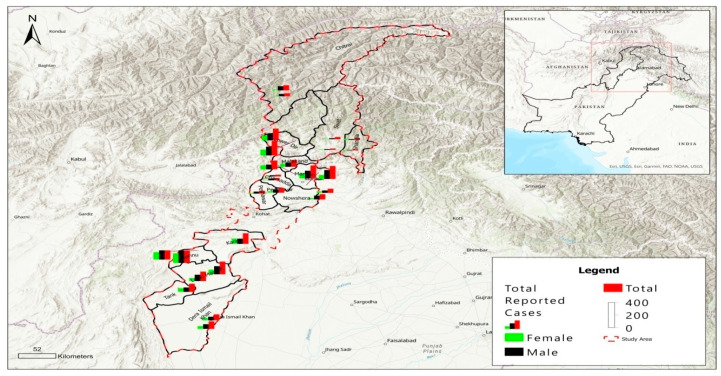
A proportional symbol map of the study area. Location of Khyber Pakhtunkhwa province is shown in the upper right corner. Gender-wise distribution of total cases reported during 2021 is scaled in colored squares.

Khyber Pakhtunkhwa province, formerly known as the North-West Frontier Province (NWFP), has an area of 101,741 km^2^ and a population of about 35.5 million, comprising 50.7% males and 49.7% of females. Sharing a border with Afghanistan and tribal areas, it consists of 35 administrative districts (Figure 1). Earthquakes attack Khyber Pakhtunkhwa province frequently because of its location in the weak tectonic zone. In addition, floods from the Indus River happen during the monsoon season every year. This province has experienced many shattering floods in the last twenty years. Of 22 serious floods recorded during 1950 to 2014, the flood in 2010 was the most devastating, affecting millions of people and households in the province [8]. In other parts of the province, particularly the mountainous areas in the north, floods occur due to torrents, landslides, glacial lake outbursts, and rapid glacial run. Forestry, mining, and agriculture are the province’s primary economic activities. Approximately 78% of marble production in Pakistan is from the province. The gross domestic product (GDP) in 2021–2022 was estimated at 1071 US dollar per capita.

### 2.2. Ethical Statement

The study has been approved by the Ethical Committee of Chemical and Life Section, Department of Zoology, Abdul Wali Khan University Mardan, Pakistan under the approval no. AWKUM-20053310.

### 2.3. Data Collection and Processing

The logic and procedure of the study is shown in Supplementary Figure S1. The clinical-epidemiological data of microscopically confirmed CL patients were obtained from health facilities of health departments across the province throughout 2021. CL patients were diagnosed at local facilities and treated and followed-up systematically. The treatment courses of CL were excluded from this study due to incompleteness of information. Other data were entered into the spreadsheets of Microsoft Excel (Windows version, 2016) for descriptive analysis. Coordinates of the districts were obtained from Google Earth Pro (version 7.3), and the average incidence of CL in each district was aligned respectively. ArcGIS (version 10.7.1) was used for spatial risk and statistical analysis. A proportional symbol map was illustrated according to the cases of risk groups in districts. A digital elevation model (DEM) was generated using the geostatistics from the link http://www.diva-gis.org/datadown (accessed on 27 January 2023), which were light detection and ranging (LiDAR) and interferometric synthetic aperture radar (IfSAR, only for Alaska) data with a grid of 10 m^2^ resolution. The exact elevation of each district was obtained by computing DEM raster output data.

### 2.4. Choropleth Map, Inverse Distance Weighting (IDW) Interpolation

A choropleth map was built by representing the number of CL cases with continuous colors in lack of fidelity among districts. Model accuracy was validated by field statistics [43]. The IDW approach was used for spatial interpolation to evaluate CL transmission. IDW is an estimation method in which a linear combination of known values of sampling points is used to predict unknown values of non-sampling points with corresponding weighted values of inverse distance [44,45]. The calculation was carried out with the settings of neighboring type, 0.5 smoothing factor, and zero angles of spatial pattern.

### 2.5. Cluster and Outlier Analysis and Hotspot Analysis

The spatial statistics tools suite in ArcGIS was used. Spatial clusters and outliers were detected by calculating a local Moran’s I value, a z-score, a pseudo *p*-value, and a code representing the cluster type for each significant feature (Anselin Local Moran’s I). Statistically significant spatial clusters of high values (hot spots) and low values (cold spots) were identified via hot spot analysis (Getis-Ord Gi*). Fixed distance band was used to ensure each feature had at least one neighbor. The Euclidean distance technique was applied to calculate the distance of the neighboring zone around the indices. Spatial weights were not row standardized [43].

### 2.6. Bayesian Geostatistical Analysis

Climate data comprising 19 bioclimatic variables which represented varied temperature and precipitation conditions with 1 (30 s) to 340 (10 min) km^2^ resolution for the years 1970–2000 were obtained from WorldClim Global Climate Data (https://www.worldclim.org/data/worldclim21.html, accessed on 23 January 2023). The data were organized in Microsoft excel sheets and R-codes were created against each variable using R-software (windows version 4.2.2) for Bayesian geostatistical analysis [41,46].

### 2.7. Statistical Analysis

The data of CL patients were inputted to spreadsheets of Microsoft Excel (Windows version, 2016) and analyzed using SPSS (v. 26, IBM Corp. in Armonk, NY, USA). Differences between groups of each risk factor were compared using chi-square tests, a nonparametric method, after testing for a normal distribution. The observed difference was considered to be statistically significant if the *p*-value was less than 0.05. Associations between variables were examined using the chi-square test of independence with a significance level of 0.05. All variables were treated as categorical.

## 3. Results

A total of 3188 microscopically confirmed CL cases were reported in Khyber Pakhtunkhwa province in Pakistan in 2021. Sixty three percent of them were males (2003/3188, 62.8%). Male patients were more abundant in all age groups and in all studied districts (*p* < 0.001) (Figure 1). The age of patients ranged from 1 to 82 years, but the majority were younger than 20 years old (1800/3188, 56.5%) (*p* < 0.001). In fact, the number of patients was observed to be inversely correlated to age groups (Table 1). Regarding clinical presentation of CL, one to five lesions were observed at different body parts of the patients. Most patients had a single lesion (1540/3188, 48.3%) (*p* < 0.001), but there were 552 patients who had more than three lesions (552/3188, 17.3%) (Table 1). The face was most frequently attacked (1151/3188, 36.1%) (*p* < 0.001), followed by the hand (1068/3188, 33.5%), foot (633/3188, 19.9%), and multiple sites (336/3188, 10.5%). The dry form of lesion (2746/3188, 86.1%) was more common than the wet form (442/3188, 13.9%) (*p* < 0.001). The type of lesion was associated with season (*p* = 0.004) and district (*p* < 0.001).

Temporally, the incidence gradually increased from October (*n* = 212) and reached a peak in February (*n* = 643), which was followed by a sharp decrease in March (*n* = 313). Overall, most of infections occurred in winter (1443/3188, 45.26%), while fewer cases were found in autumn (446/3188, 13.98%) (*p* < 0.001) (Table 1). The seasonal trend was particularly apparent in the southern region (Table 2). However, in northern districts no patient was identified in Chitral in summer, although five male patients were reported in autumn. In Dir Lower, the fewest CL cases were recorded in spring (2/421, 0.5%), in contrast to Malakand, which had the highest number of infections in spring (107/250, 42.8%). The seasonal difference of cases in Swat was not significant. In the central region, there were almost as many patients in winter as in summer in Mardan. CL was least seen in Noshehra but most prevalent in Peshawar in spring. Charsadda did not report any patients in summer and autumn. In addition, the gender of patients was associated with season (*p* < 0.001). The sex ratios were 1.4, 1.8, 1.9, and 2.4 males/female for winter, spring, summer, and autumn, respectively. Spatially, CL was more prevalent in southern region (1501/3188, 47.08%) compared with northern (865/3188, 27.13%) and central regions (822/3188, 25.78%) (*p* < 0.001) (Table 1). The highest incidence of CL was observed in the city of Kaka Khal (*n* = 370) in the Bannu district, followed by the city of Domain (*n* = 263) and the city of Khal (*n* = 232) in the Dir Lower district. In total, 633 (633/3188, 19.9%) patients were from the Bannu district, 421 (421/3188, 13.2%) patients were from the Dir Lower district, and 411 (411/3188, 12.9%) patients were from the Mardan district.

**Figure 2:**
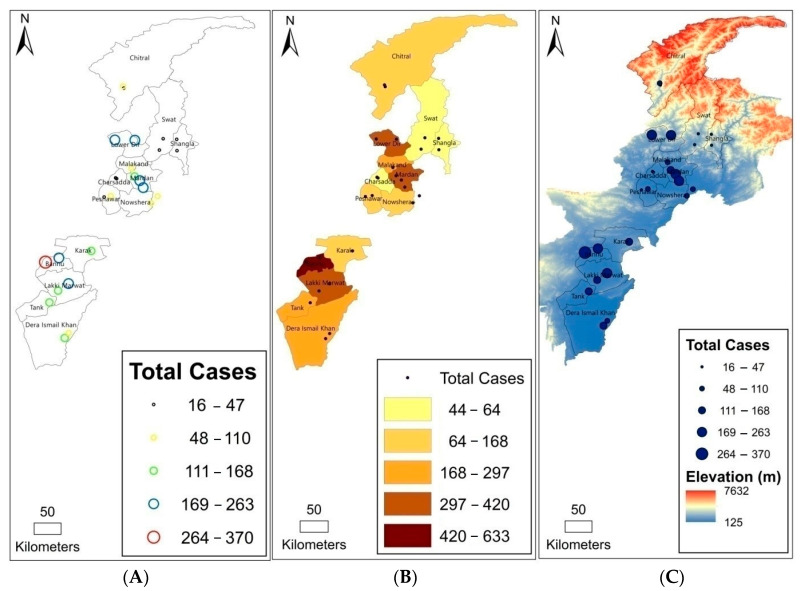
(**A**) Cutaneous leishmaniasis (CL) cases in each city, showing the highest and lowest incidences across the province; (**B**) a choropleth map showing district-based CL cases; (**C**) a digital elevation model (DEM) map illustrating the occurrence of CL at different altitudes.

CL was observed in 24 cities in 12 districts of Khyber Pakhtunkhwa, showing transmission of CL across the study area. The incidence of CL in each city was tagged to provide a visual concept of prevalence across the province (Figure 2A). The choropleth map showed the darkest color in Bannu in the southern region, highlighting the highest average case number in the district. The next dark color filled in Dir Lower in the northern region, Mardan in the central region, and Lakki Marwat in the southern region. The lightest color was used for Swat in the northern region, representing the fewest average cases (Figure 2B). The DEM map revealed that CL was found at altitudes of 125 to 7632 m, but incidence decreased with high altitudes. Cases were mostly distributed at low altitudes in the south (Figure 2C). An elevation range of 946 to 1977 m was identified to be associated with the highest occurrence of *Leishmania* infection.

**Figure 3:**
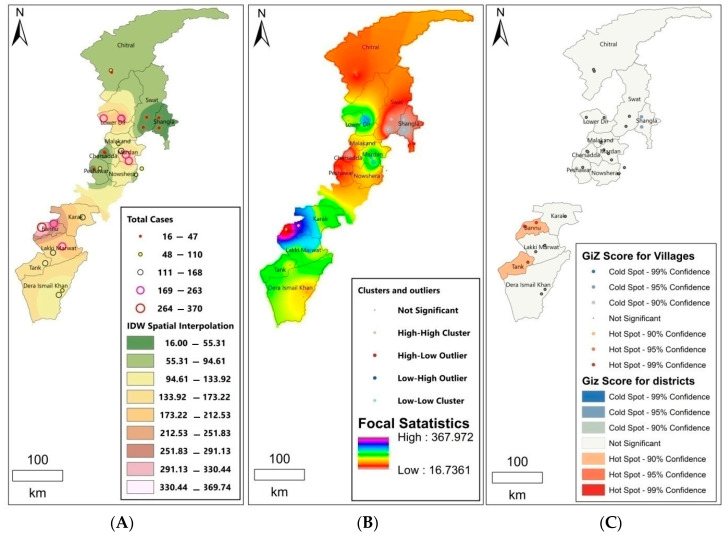
(**A**) Cutaneous leishmaniasis (CL) transmission analyzed by inverse distance weighting (IDW) interpolation; (**B**) focal statistics with cluster and outlier analysis; (**C**) hotspot analysis.

IDW interpolation was applied to analyze the transmission patterns and therefore predict the future epidemic risk of CL. Threat focus areas were presumed to be located in the vicinity of the current CL-prevalent districts, including Bannu (IDW Spatial interpolation: 330.44–369.74), Dir Lower (IDW Spatial interpolation: 291.13–330.44), and Mardan (IDW Spatial interpolation: 251.83–291.13) (Figure 3A). The results of IDW showed high and low endemic areas across the province, with the highest value of focal statistics of 367.972 and the lowest value of 16.7361 (Figure 3B). Cluster and outlier analysis indicated Bannu (99% confidence interval, *p* < 0.05), Dir lower (95% confidence interval, *p* < 0.05), and Mardan (95% confidence interval, *p* < 0.05) as high cluster districts, as the other districts were not significant (Figure 3B). Hotspot analysis identified Bannu and Tank as significantly high clusters (hotspots) and Shangla as a low cluster district (coldspot) (Figure 3C).

Bayesian geostatistical analysis showed high temperature, low altitude, and annual precipitation were associated with the predicted highest number of cases in Kaka Khel (Bannu, *p* = 0.001), Khal (Dir Lower, *p* = 0.001), and Toro (Mardan, *p* = 0.001). Humidity and varied seasonal precipitation were associated with CL less affected areas such as Swat and Chitral.

## 4. Discussion

CL has been prevalent and is still expanding its frontier in Pakistan, including Khyber Pakhtunkhwa, a northern province bordering Afghanistan and Baluchistan [35,47]. One of the major outbreaks in that area occurred in the Timer Camp for Afghan refugees in 1997, indicating the spread of the disease due to the migration of the population [22]. However, transmission and geological expansion of CL were rather difficult to identify because of the diversity of disease incubation periods. This cross-sectional study was conducted to explore the spatial and temporal distribution of CL in Khyber Pakhtunkhwa. Clinico-epidemiological data of 3188 patients were collected from local health facilities throughout 2021. The seasonal occurrence of CL was analyzed statistically, and the geographical distribution, focal transmission, and hot spots were investigated using GIS tools.

Of 3188 patients reported in 2021, nearly 20% (633/3188, 19.9%) came from Bannu. This may be due to the high temperature and population density in Bannu compared with other districts in the province. Higher incidence of CL has been observed in areas with high population density and unhygienic environments [15]. Unsatisfactory housing conditions, such as cracked mud walls, dampness, and darkness as an indicator of poverty also increased the risk of leishmaniasis [48,49]. Dir Lower was found to be the second most affected district in the study. One of the reasons could be attributed to the inflow of Afghan refugees since 1970, but CL became even widespread among local inhabitants in combination with the abundance of sandflies in the district [50,51,52,53,54]. Moreover, the northern region was famous for various types of tourism and business activities. Migration of people between non-endemic and endemic areas would play a key role in the spread of CL [55]. Similar differences in prevalence between districts have been demonstrated in previous reports [5,17,32].

Sandfly species of *Phlebotomus* and *Sergentomyia* genera are abundant in Khyber Pakhtunkhwa [56,57,58,59,60]. Although there have been no conclusive reports to clarify the pathogen-vector association between *Leishmania* and sandflies in Pakistan, *Phlebotomus papatasi* and *Phlebotomus sergenti*, the two most common species in several districts, are considered to be vectors of CL, while *Phlebotomus major* and *Phlebotomus hindustanicus* are suspected vectors of VL [56]. Studies in Iran have identified *Ph. papatasi* as a vector of *Leishmania major* [61,62], and *Ph. sergenti* has been shown to transmit *Leishmania tropica* in Morocco [63]. *Phlebotomus caucasicus* and *Phlebotomus salehi* were also indicated as potential vectors of CL in Middle East countries [64,65,66]. Other sandflies found in Khyber Pakhtunkhwa included *Phlebotomus salengensis*, *Phlebotomus andrejevi*, *Phlebotomus kazeroni*, *Phlebotomus bergeroti*, *Phlebotomus ansari*, *Phlebotomus alexandri*, *Sergentomyia babu*, *Sergentomyia dentata*, *Sergentomyia baghdadis*, *Sergentomyia bailyi*, *Sergentomyia hospittii*, *Sergentomyia montana*, *Sergentomyia grekovi*, *Sergentomyia hodgsoni*, *Sergentomyia turkistnica*, *Sergentomyia tiberidis*, *Sergentomyia theodori*, *Sergentomyia sumbarica*, *Sergentomyia dreyfussi turkestanica*, and *Sergentomyia fallax afghanica* [57,58,59]. On the other hand, records about reservoir hosts of CL are scarce in Pakistan. Great gerbils *(Rhombomys opimus*) were the primary natural hosts of *Leishmania* spp. in many countries in Central Asia [66]. The role great gerbils and other small mammals, e.g., *Meriones erythrourus*, played in the transmission cycle of *Leishmania* in Khyber Pakhtunkhwa remains to be investigated.

In our study, males appeared to be more affected than females. This may be due to the fact that females cover themselves and remain indoors most of the time in Pakistan. Males were more exposed to the bites of sandflies as they went for labor with their faces, shoulders, and hands uncovered. The association between season and gender further supported the hypothesis. The sex ratios of CL patients were 1.4, 1.8, 1.9, and 2.4 males/females for winter, spring, summer, and autumn, respectively (853/590, 426/240, 411/222, and 313/133). The low temperature in winter made both females and males stay indoors at night, when sandflies were seeking bloodmeals actively, while the warmer weather in summer would allow male workers to go outdoors after sunset. CL infection was observed across all ages, but children and young people under the age of 20 years were most vulnerable. Playing outside in the shade of trees or near humid ground surfaces where sandflies bred placed children at high risk of vector bites. Their immature immune systems also led to increasing susceptibility to infection [67,68,69].

Our data indicated that CL was more prevalent in winter, especially in the southern region, although other studies identified a high incidence in spring [32,70,71]. The occurrence of CL was in fact associated with the fluctuation of sandflies in the rainy season [72]. In the northern region, frontal cloud bands result in precipitation in winter. Heavy thunderstorms sometimes happen in Chitral in spring. Dir Lower is one of the wettest districts in Pakistan with an annual rainfall of 1473.2 mm, of which 400 mm are brought by the monsoon in summer. Almost twice that amount takes place during December to April, showing a bimodal rainfall regime. CL infection was mostly reported in winter (*n* = 221) and summer (*n* = 131), echoing the precipitation pattern.

Clinically, most of the patients in the study had a single facial lesion, consistent with previous findings [73,74]. The face was the body part most exposed to sandfly bites. The situation was worsened if people did not carefully take preventive measures, such as wearing repellents, using bed nets, and avoiding outdoor activities during the time sandflies were active [48]. Lesions over five centimeters were noticed on seven patients. The size of lesion was associated with limited access to medical care [48,75,76]. As a result, patients with low socio-economic status suffering from CL might have to live with facial disfigurements in developing countries. On the other hand, we also found the type of lesion was significantly associated with temporal and spatial factors (season and districts, *p* = 0.004 and *p* < 0.001, respectively). The wet type of lesion was chiefly caused by *L. major* and had a shorter incubation period (two weeks). This type of CL, also referred as zoonotic CL, was identified to be prevalent at low elevations in arid and semi-arid areas in Pakistan [3]. The dry form, caused by *L. tropica* and also called anthroponotic CL, was transmitted at high elevations (500–2837 m) [3]. The ratio of patients with dry to wet form CL was lowest in autumn (360/86, 4.2), while it was higher in winter and spring (1260/183 and 582/84, 6.9 and 6.9, respectively). One limitation of the study was the lack of species identification for the etiological agents causing the observed lesions due to limited resources in the local health facilities and the study design. However, previous reports have shown that *L. tropica* and *L. major* were responsible for most CL cases in Khyber Pakhtunkhwa [3,23,48]. Other pathogens, such as *Leishmania infantum*, have been detected in patients, but the ratios were quite low [47]. The *Leishmania* species causing CL in Khyber Pakhtunkhwa and their dynamics will be further investigated in the future.

The spatial pattern of CL patients was presented with a choropleth map. The continuous color represented the abundance of patients varied in districts. There were more patients reported in Bannu, in contrast to fewer patients reported in Swat and Charsadda. Visualization of case distribution would allow public health sectors to easily assess regional severity of the infectious disease [77]. Meanwhile, the vertical occurrence of CL was illustrated by a DEM map. CL patients were distributed between the elevations of 125 to 7632 m, although most of the patients lived at 946 to 1977 m. The vertical dissemination of CL would be restricted by the activities of sandflies, which prefer warm weather at low altitudes.

The transmission of CL was predicted using the IDW method based on the hypothesis that the chance of being infected decreased with growing distance to an existing case, as sandflies are weak fliers. The CL occurrence of neighborhood locations close to the cases was estimated by spatial interpolation, as both locations shared similar characteristics, including land use, lifestyle of the inhabitants, and breeding sources of sandflies. The interpolation could provide public health sectors with a reference for disease prevention [77]. Risk of CL was further assessed using cluster and outlier analysis as well as hotspot analysis. Apparent clusters were identified in areas around the current CL-prevalent districts including Bannu and Dir Lower. Hotspot analysis also identified Bannu as an area of high clustering (hotspots). Previous studies have described household clusters and climate as the main factors affecting transmission of CL [78,79]. Bayesian geostatistical analysis was then applied to evaluate the effects of environmental and climatic variables on the spatial distribution of the disease. The results indicated a high incidence in the southern region was associated with low altitudes, high temperature, and increasing annual precipitation. Warm weather and precipitation have been shown to facilitate the transmission of CL, as the development of both sandflies and *Leishmania* accelerate at high temperatures, and precipitation results in more suitable breeding sites for sandflies [41].

Although the WHO has taken action in an attempt to control CL in endemic areas, CL remains a major public health problem in Pakistan [80,81]. GIS has been extensively applied to describe spatial patterns and shows great promise in research and surveillance of diseases. This study utilized GIS tools to illustrate the horizontal and vertical distribution of CL in Khyber Pakhtunkhwa, Pakistan. Transmission, risk, and hotspots of the disease were evaluated and presented visually. Clinical presentations as well as risk factors including gender, age, season, and region were analyzed statistically. Additionally, Bayesian inference was used in geostatistics to explore the relationship between CL occurrence and climatic variables. The results could help public health sectors to establish prevention and control strategies for upcoming CL epidemics. For instance, health education especially targeting risk groups could be provided. Combined with population data, intervention could be prioritized in areas with the highest disease risk, e.g., the Bannu district, and resources could be distributed to those in need. Efforts for disease prevention and vector control could be made before the epidemic season of each district specifically according to the results of temporal analysis. Public health workers should help to raise the awareness of clinicians and residents in hotspots, thus allowing infected patients to receive treatment earlier. When climate factors favor the growth of vectors, special attention has to be paid to surveillance, and sufficient medical supplies must be prepared for potential outbreaks. Moreover, from a worldwide perspective, this study could be beneficial for global CL control.

## 5. Conclusions

The study presented an overview of CL in Khyber Pakhtunkhwa in Pakistan. The majority of patients were males or individuals under the age of 20 years. Infection mostly occurred in the southern region in winter. GIS tools were used to visualize the spatial patterns of the disease, and high clusters were found in Bannu, Dir Lower, and Mardan. Identification of risk areas would be helpful for public health sectors to predict future epidemics and implement corresponding interventions.

## Figures and Tables

**Table 1:** Characteristics of cutaneous leishmaniasis patients in Khyber Pakhtunkhwa, Pakistan, 2021.

Variable	Description	Case no.	Percentage (%)	*p* Value
Gender	Male	2003	62.8	<0.001
	Female	1185	37.2	
Age group	1–20	1800	56.5	<0.001
	21–40	872	27.4	
	41–60	409	12.8	
	>60	107	3.4	
Season *	Winter	1443	45.3	<0.001
	Spring	666	20.9	
	Summer	633	19.9	
	Autumn	446	14.0	
Lesion site	Face	1151	36.1	<0.001
	Hand	1068	33.5	
	Foot	633	19.9	
	Multiple	336	10.5	
Lesion type	Dry	2746	86.1	<0.001
	Wet	442	13.9	
Lesion number	One	1540	48.3	<0.001
	Two	626	19.6	
	Three	470	14.7	
	>Three	552	17.3	
Northern region	Chitral	150	4.7	<0.001
	Dir Lower	421	13.2	
	Malakand	250	7.8	
	Swat	44	1.4	
Central region	Mardan	411	12.9	<0.001
	Charsadda	64	2.0	
	Noshehra	228	7.2	
	Peshawar	119	3.7	
Southern region	Bannu	633	19.9	<0.001
	DI Khan	215	6.7	
	Lakki Marwat	354	11.1	
	Tank	299	9.4	

* Winter: December ~ February; spring: March ~ May; summer: June ~ September; autumn: October ~ November.

**Table 2:** Seasonal and gender-wise prevalence of CL in Khyber Pakhtunkhwa, 2021.

District	Season	*n*	%	*p* Value	Female			Male		
					*n*	%	*p* Value	*n*	%	*p* Value
Bannu	Winter	217	34.3	<0.001	89	38.9	< 0.001	128	31.7	<0.001
	Spring	172	27.2		67	29.3		105	26.0	
	Summer	170	26.9		51	22.3		119	29.5	
	Autumn	74	11.7		22	9.6		52	12.9	
Charsadda	Winter	37	57.8	0.211	7	41.2	0.467	30	63.8	0.058
	Spring	27	42.2		10	58.8		17	36.2	
	Summer	0	0.0		0	0.0		0	0.0	
	Autumn	0	0.0		0	0.0		0	0.0	
Chitral	Winter	102	68.0	<0.001	14	77.8	0.018	88	66.7	<0.001
	Spring	43	28.7		4	22.2		39	29.5	
	Summer	0	0.0		0	0.0		0	0.0	
	Autumn	5	3.3		0	0.0		5	3.8	
DI Khan	Winter	96	44.7	< 0.001	41	47.1	<0.001	55	43.0	<0.001
	Spring	47	21.9		16	18.4		31	24.2	
	Summer	28	13.0		12	13.8		16	12.5	
	Autumn	44	20.5		18	20.7		26	20.3	
Dir Lower	Winter	221	52.5	< 0.001	95	55.2	<0.001	126	50.6	<0.001
	Spring	2	0.5		2	1.2		0	0.0	
	Summer	131	31.1		57	33.1		74	29.7	
	Autumn	67	15.9		18	10.5		49	19.7	
Lakki Marwat	Winter	139	39.3	<0.001	49	39.2	0.002	90	39.3	<0.001
	Spring	78	22.0		30	24.0		48	21.0	
	Summer	64	18.1		22	17.6		42	18.3	
	Autumn	73	20.6		24	19.2		49	21.4	
Malakand	Winter	69	27.6	<0.001	36	33.0	< 0.001	33	23.4	<0.001
	Spring	107	42.8		43	39.4		64	45.4	
	Summer	43	17.2		13	11.9		30	21.3	
	Autumn	31	12.4		17	15.6		14	9.9	
Mardan	Winter	134	32.6	<0.001	56	36.6	< 0.001	78	30.2	<0.001
	Spring	89	21.7		40	26.1		49	19.0	
	Summer	130	31.6		43	28.1		87	33.7	
	Autumn	58	14.1		14	9.2		44	17.1	
Noshehra	Winter	86	37.7	<0.001	40	43.5	0.001	46	33.8	<0.001
	Spring	34	14.9		20	21.7		14	10.3	
	Summer	42	18.4		16	17.4		26	19.1	
	Autumn	66	28.9		16	17.4		50	36.8	
Peshawar	Winter	30	25.2	<0.001	2	66.7	0.564	28	24.1	<0.001
	Spring	57	47.9		1	33.3		56	48.3	
	Summer	11	9.2		0	0.0		11	9.5	
	Autumn	21	17.6		0	0.0		21	18.1	
Swat	Winter	13	29.5	0.436	6	24.0	0.706	7	36.8	0.443
	Spring	10	22.7		7	28.0		3	15.8	
	Summer	14	31.8		8	32.0		6	31.6	
	Autumn	7	15.9		4	16.0		3	15.8	
Tank	Winter	299	100.0		155	100.0		144	100.0	
	Spring	0	0.0		0	0.0		0	0.0	
	Summer	0	0.0		0	0.0		0	0.0	
	Autumn	0	0.0		0	0		0	0.0	

## Data Availability

Not applicable.

## Supplementary Materials

The following supporting information can be downloaded at: https://www.mdpi.com/article/10.3390/tropicalmed8020128/s1, Figure S1: Flowchart of the study.
