# Supplementary figures and images for: Distribution and Risk of Cutaneous Leishmaniasis in Khyber Pakhtunkhwa, Pakistan
Source: Trop Med Infect Dis. 2023 Feb 20;8(2):128. doi: 10.3390/tropicalmed8020128 (PMC9962270; doi:10.3390/tropicalmed8020128)

## Supplementary Figure S1.

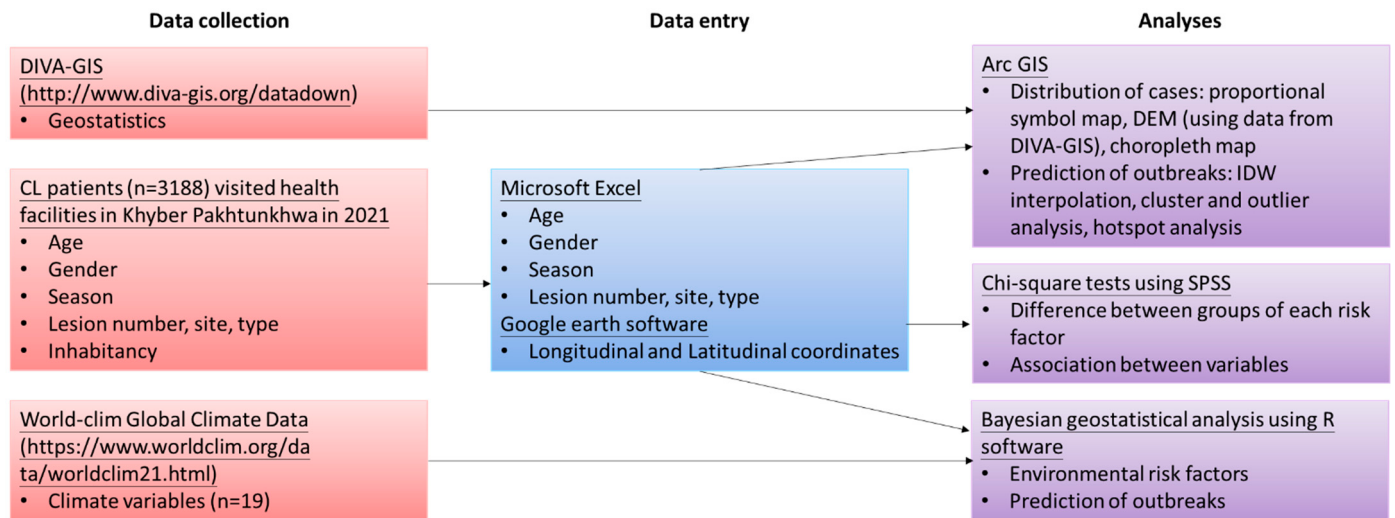

**Figure S1.** Flowchart of the study.

Supplement: Supplementary file 1 [file tropicalmed-08-00128-s001.zip › tropicalmed-2198817-supplementary.pdf]
